# Generation of Augmented Capillary Network Optical Coherence Tomography Image Data of Human Skin for Deep Learning and Capillary Segmentation

**DOI:** 10.3390/diagnostics11040685

**Published:** 2021-04-10

**Authors:** Bitewulign Kassa Mekonnen, Tung-Han Hsieh, Dian-Fu Tsai, Shien-Kuei Liaw, Fu-Liang Yang, Sheng-Lung Huang

**Affiliations:** 1 Graduate Institute of Electro-Optical Engineering, National Taiwan University of Science and Technology, No. 43, Keelung Rd., Sec. 4, Da’an Dist., Taipei City 10607, Taiwan; bitkassa2006@gmail.com (B.K.M.); skliaw@mail.ntust.edu.tw (S.-K.L.); 2 Research Center for Applied Sciences, Academia Sinica, No. 128, Academia Rd., Sec. 2, Nankang, Taipei City 11529, Taiwan; a28238341a@gmail.com (D.-F.T.); flyang@sinica.edu.tw (F.-L.Y.); 3 Department of Electrical Engineering, National Taiwan University of Science and Technology, No. 43, Keelung Rd., Sec. 4, Da’an Dist., Taipei City 10607, Taiwan; 4 Graduate Institute of Photonics and Optoelectronics, National Taiwan University, No. 1, Sec. 4, Roosevelt Rd., Taipei City 10617, Taiwan; shuang@ntu.edu.tw

**Keywords:** full-field optical coherence tomography, skin capillary segmentation, augmented dataset generation, deep learning, U-Net, image binarization

## Abstract

The segmentation of capillaries in human skin in full-field optical coherence tomography (FF-OCT) images plays a vital role in clinical applications. Recent advances in deep learning techniques have demonstrated a state-of-the-art level of accuracy for the task of automatic medical image segmentation. However, a gigantic amount of annotated data is required for the successful training of deep learning models, which demands a great deal of effort and is costly. To overcome this fundamental problem, an automatic simulation algorithm to generate OCT-like skin image data with augmented capillary networks (ACNs) in a three-dimensional volume (which we called the ACN data) is presented. This algorithm simultaneously acquires augmented FF-OCT and corresponding ground truth images of capillary structures, in which potential functions are introduced to conduct the capillary pathways, and the two-dimensional Gaussian function is utilized to mimic the brightness reflected by capillary blood flow seen in real OCT data. To assess the quality of the ACN data, a U-Net deep learning model was trained by the ACN data and then tested on real in vivo FF-OCT human skin images for capillary segmentation. With properly designed data binarization for predicted image frames, the testing result of real FF-OCT data with respect to the ground truth achieved high scores in performance metrics. This demonstrates that the proposed algorithm is capable of generating ACN data that can imitate real FF-OCT skin images of capillary networks for use in research and deep learning, and that the model for capillary segmentation could be of wide benefit in clinical and biomedical applications.

## 1. Introduction

Blood vessels make up a complex system that plays a prominent role in homogenization processes, such as angiogenesis, fluid and solute balance, thrombosis, perfusion/oxygenation, and blood pressure [1,2]. The blood vessel system is an important prognostic indicator of many clinical outcomes in various areas of medicine, such as neurosurgery [3], laryngology, oncology [4], and ophthalmology. The system comprises arteries, capillaries, and venous networks [2,5]. Capillary networks are microvascular networks (the smallest vessels), only 7–10 µm in diameter; they are present in all human body tissues and are of a size sufficient to transport red blood cells [6,7].

To date, numerous three-dimensional (3D) optical spectroscopy imaging modalities have attracted attention for in vivo human skin capillary imaging in clinical studies. These modalities include computed tomography angiography (CTA), magnetic resonance imaging (MRI) [8,9], ultrasound imaging [10], micro-computed tomography (MCT) [11], confocal laser scanning microscopy (CLSM) [12], and optical coherence tomography (OCT), all of which are able to visualize arteries, veins, and capillaries. Among these technologies, OCT has, in the past few years, been attracting the attention of many researchers for disease diagnosis and clinical treatment. OCT provides real-time, contactless, and noninvasive optical 3D scans of in vivo tissue at high resolution. Typically, Mirau-based full-field optical coherence tomography (FF-OCT) [13] produces high-definition in vivo skin images with a spatial resolution of 0.9 µm transversely and 0.51 µm longitudinally in en face and cross-sectional views by using a Ce3+:YAG double-clad crystal fiber light source.

In dermatology, in addition to being used for the direct detection of various skin cancers, such as basal cell carcinoma [14,15], melanoma, and squamous cell carcinoma [16], OCT provided an opportunity for the in-depth study of lesion development. Research has found that the presence of skin cancers often coincides with an abnormal proliferation of the surrounding blood vessel system [4,17,18], which delivers oxygen and nutrients that support the growth of the malignancies. FF-OCT provides the capability of resolving vessels and capillaries in high-resolution images [14,19,20], which could help physicians track the maturity of tumors and the effect of drug delivery, allowing them to examine the evolution of the nearby angiogenesis. Past studies [4,21,22] have also demonstrated that inhibiting angiogenesis is a viable approach for cancer therapy. Moreover, real-time capillary network identification may help physicians make proper and timely decisions during clinical treatment. For example, during and after Mohs micrographic surgery for skin tumor removal, the ability to precisely localize surrounding capillaries could successfully prevent metastasis, minimize the damage of nearby vessels and capillaries, and reduce bleeding [23,24]. In addition, the rapid and noninvasive identification and quantification of capillaries are clinically useful for accurately estimating carcinoma morphology and size variation among capillaries. This capability of FF-OCT provides clinicians with the advantage of being able to precisely examine capillary blood flow, skin condition, and function, allowing them to detect abnormalities [25,26,27].

Capillary segmentation from FF-OCT data may not be a trivial task. Traditionally, people examined the entire volume of human skin in en face FF-OCT images slice by slice to identify and manually annotate the capillaries. However, capillaries are not often easily distinguishable in an FF-OCT image because of their low contrast relative to the background. Due to advances and improvements in the camera, FF-OCT can rapidly produce hundreds of in-depth images. If all volumetric images had to be examined and labeled manually by dermatologists, it would take an extremely long time to handle all the image data. Thus, extracting and examining capillaries in FF-OCT images of human skin in vivo is a major challenge. To address this challenge, it is necessary to build an automatic segmentation and tracking method for capillaries.

There are a few studies being conducted for this task in the analysis of in vivo human skin FF-OCT image data. Lee et al. [14,15] developed a short-time robust principal component analysis (RPCA) algorithm for capillary segmentation. The basic idea is to separate fast-moving red blood cells along the capillaries from the nearly static tissue background. Although this method of detecting capillaries outperforms the traditional RPCA method, the algorithm is vulnerable to the scarcity of labeled data images. Enfield et al. [28] presented capillary extraction from volumes of in vivo OCT images of human volar forearm using correlation mapping optical coherence tomography (cmOCT) techniques. Currently, benefitting from recent advances in deep learning techniques in medical image preprocessing, capillary segmentation via supervised deep learning has become a hot topic for many researchers [29,30], who are using it for automatic and accurate segmentation. Prior to training a deep learning model, a large, representative, and high-quality labeled dataset [31] (the training data) with the ground truth is required to obtain state-of-the-art performance. Again, the labeling/annotating tasks to prepare the raw images of the training data are often performed manually by specialists via visual evaluation [32], which depends on the person’s skill, and is very laborious, expensive, and time-consuming. Thus, scarce and weakly labeled (annotated) FF-OCT images create a major challenge in training deep convolutional neural networks [33] to develop computer-aided diagnosis. To address this problem, we attempted to develop a simulation algorithm for the automatic generation of ground truth and the corresponding augmented OCT input images simultaneously. 

In the past, few simulation algorithms were proposed to construct capillary networks. Van Leeuwen et al. [34,35] presented an algorithm to generate 3D artificial capillary networks that have very similar natures to realistic magnetic resonance imaging (MRI). Xion et al. [36] constructed 3D deformable artery modeling to simulate blood flow and vessel wall dynamics from medical images. Flyckt et al. [37,38] introduced a blood flow model based on a bioheat model. The Lagendijk model and discrete vasculature (DIVA) were proposed based on the thermal model approach for in vivo human eye and orbit images. Kotte et al. [34] presented a DIVA thermal model to extend single vessel segment modeling [39]. Although these algorithms revealed good arterial and venous networks results, the distribution of the networks showed end-to-end connections. In addition, the networks also shrink in both diameter and length for the branch, where larger diameters occur in trunk arteries or veins. However, our target is the capillaries. Looking closely at FF-OCT images, any branch of a capillary network has almost the same diameter segmentally distributed across the en face images. Consequently, this study presents a number of modifications to simulate capillary networks.

This paper has two major objectives: (i) to develop a simulation algorithm for automatic generation of augmented capillary network (ACN) patterns of imitated OCT data comprising both the ground truth and corresponding input images (which we call the ACN data), and (ii) to develop a deep learning technique for capillary segmentation based on the ACN data to evaluate the feasibility of our proposed algorithm and for practical use in research and clinical applications. The validity of the ACN data is assessed by testing the model on real FF-OCT image data. Our approach consists of the following steps: (i) Model the capillary pathways by using the potential function and parametric fitting. (ii) Apply two-dimensional Gaussian spots along the capillary pathways to mimic the lightened visualization of capillaries found in real OCT data for input images and define the diameter of augmented capillaries for the ground truth. (iii) Randomly truncate the capillary pathways into segments. (iv) Add Gaussian white noise with speckles as the background to create input images that imitate real FF-OCT skin images. (vi) Stack the generated 2D images (some contain augmented capillary pathways, and some are empty backgrounds) into volumetric images with dimensions of 128 × 128 × 64 pixels to form the ACN data. (vii) Train a deep learning model based on the ACN data to evaluate its quality. Real in vivo volumetric human skin images are tested for the assessment. Finally, the proper way to binarize the predicted results in order to compare with the ground truth is discussed.

## 2. Materials and Methods

**Figure 1:**
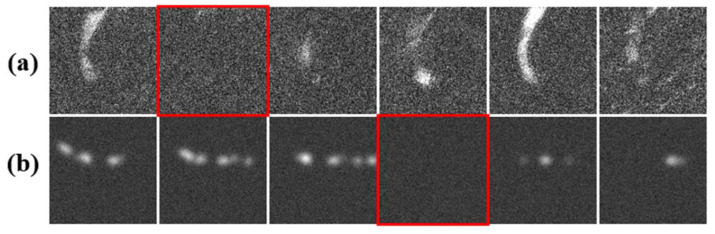
(**a**) Six successive frames of en face FF-OCT images of capillaries in human skin in vivo, with pure background layer in between (red square) indicating no red blood cell flow at that instance. (**b**) Six successive frames of augmented en face image volume to simulate capillary network, with pure background frame randomly inserted to imitate real case in (**a**).

Figure 1a presents six successive frames of en face FF-OCT images of human skin in vivo, showing a capillary network system. In reality, capillaries are visible only when red blood cells are passing through due to their high reflection rate. On a short time scale, red blood cells passing by are not perfectly continuous. Due to the extremely fast image grabbing time of FF-OCT (typically 210 µs per frame [13]), each image frame corresponds to the visualization of a time instance. As a result, the capillaries seen in each OCT image frame may not be continuous; instead, they are often fragmented from one frame to another. As a result, to figure out the overall capillary network, one has to investigate the fragmented capillaries in consecutive frames. In order to imitate reality, the algorithm described below was developed to generate an augmented image volume of OCT-like images with artificial pathways, as with the capillary network system (see Figure 1b). 

### 2.1. Governing Equations for Capillary Pathway Modeling

**Figure 2:**
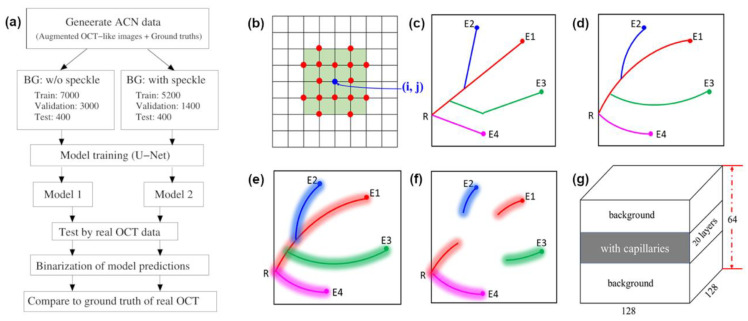
(**a**) Flowchart of this work. (**b**) Choice of growing directions of forming pathway. One of the points labeled by red dots closest to the steepest decent direction was selected to be next to the blue point, which belongs to the forming pathway. (**c**) Formed pathway network under total potential field, where R, and E1, E2, E3, E4 represent root point and four sampled end points, respectively. Note that steepest direction often leads to a straight-line pathway. (**d**) Straight and sharp folding pathways smoothed by parametric fitting curves. (**e**) Spreading of pathway network using Gaussian spots to mimic capillaries seen in real OCT images. (**f**) Random truncation of pathway network. (**g**) Layout of augmented target volumetric images used for training deep learning model for assessment.

A flowchart of this work is presented in Figure 2a, and the procedure for generating the ACN data is presented in Figure 2b–g. In this work, the governing potential functions for modeling the capillary pathways are adopted from Van Leeuwen et al. [35] with a few modifications. The potential functions are introduced to guide the pathways of capillaries and form the network. The construction of the capillary networks was performed in the two-dimensional working domain with size $L_{x}L_{y}$, where $L_{x}$ and $L_{y}$ are the width and height in number of pixels of the working domain, respectively. 

The idea of modeling capillary pathways was borrowed from the phenomenon of a river basin flowing through ravines toward the sink. A “root point” was randomly selected at the boundary of the working domain, serving as the “sink” of the river basin. The potential field, called the “root potential”, was introduced to guide the river flowing toward the root point [35]:

(1) Uroot=α [(x−x0)2+ (y−y0)2]14 

where $x_{0}$ and $y_{0}$ represent the coordinates of the root point, and the positive constant α is the strength factor. Next, a few end points were randomly generated inside the working domain. Starting from one end point, the pathway was drawn toward the root point along the direction of steepest descent of the root potential in Equation (1). The choice of directions to grow the pathway is illustrated in Figure 2b. As with a newly formed river valley, the newly formed pathway also contributes a potential field, which slightly modifies the original root potential to establish a small attraction field toward this pathway. The potential field contributed by the capillary pathway is expressed as follows [35]:

(2) Ui= β∑j[(x−xi,j)2+ (y−yi,j)2]γ2

where *i* denotes the label of the newly formed pathway, $\left(x_{i,j},\;y_{i,j}\right)$ denotes the coordinates of the list of points *j* along pathway *i*, and β and γ are dimensionless negative constants, which define the strength of the attracting field and the attracting range around the pathway, respectively. Thus, the total potential is the sum of the root potential and the potential contributed by pathway *i*. As a result, whenever nearby pathways form after pathway *i*, the steepest descent of the total potential will guide its direction toward the root point and pathway *i.* If the nearby pathway is close enough to pathway *i*, such that attraction toward pathway *i* is larger, it might attach to pathway *i* to form a branch instead of ending at the root point. Repeating the whole procedure for the other end points one by one, finally a randomly formed pathway network in the working domain was produced, which was utilized to model the en face FF-OCT images of the capillary network inside the skin tissue. Figure 2c shows the construction procedure of the pathway network for one root point and four end points, in which two of the end points form branches of pathway 1.

The pathway branches of the capillary network formed by the potential functions were guided along the direction of steepest descent of total potential. Thus, they usually presented linear segments that were far from ideal for representing realistic capillary routes. Therefore, parametric fitting was introduced to bend the straight segments and smooth the sharply folded pathways in order to mimic a realistic case (see Figure 2d). In our implementation, the third-order polynomial was applied for pathway smoothing. The characteristic polynomials are defined as follows.

(3) $$x=a_{3}y^{3}+\;a_{2}y^{2}+\;a_{1}y+\;a_{0}$$

(4) $$y=b_{3}x^{3}+\;b_{2}x^{2}+\;b_{1}x+\;b_{0}$$

**Figure 3:**
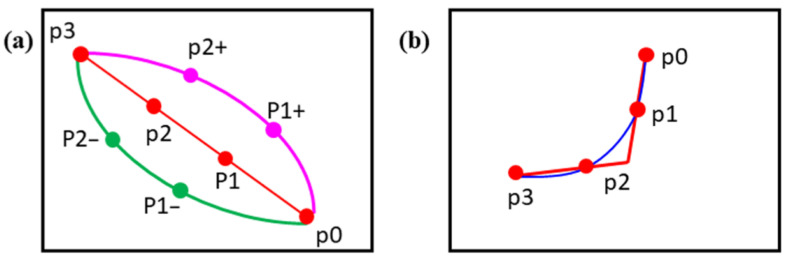
(**a**) Process of parametric curve bending for straight pathway. Straight segment passing through p0, p1, p2, and p3 is original pathway. Points (p1+, p2+) and (p1–, p2–) are those after applying slightly right and left shifts for (p1, p2), respectively. Parametric curve fitting was performed on either (p0, p1+, p2+, p3) or (p0, p1–, p2–, p3) to form curved bending pathway. (**b**) Smoothing process for sharply folded pathway. Red line and blue curve represent pathway before and after parametric curve fitting, and red points are used to solve parametric parameters.

Four points along the pathway of the branch—the first one ($x_{0},\;y_{0}$), the one at the one-third position ($x_{1},\;y_{1}$), the one at the two-thirds position ($x_{2},\;y_{2}$), and the last one ($x_{3},\;y_{3}$)—were selected and substituted into Equation (3) or (4) to solve for coefficients $\left(a_{0},\;a_{1},\;a_{2},\;a_{3}\right)$ or $\left(b_{0},\;b_{1},\;b_{2},\;b_{3}\right)$, respectively. Depending on the overall shape of the branch, either Equation (3) or (4) was used to make sure that the corresponding variables (*x* or *y*) were monotonically changing along the pathway. The resulting polynomial was then used to replace the original branch to represent the curved pathway. Figure 3 shows the bending and smoothing process of the straight segments. For pathways that were separated distantly, the steepest decent direction usually connected the end points to the root point directly. In this case, the selected middle points ($x_{1},\;y_{1}$) and ($x_{2},\;y_{2}$) of the segment were shifted slightly in order to get the curved bending segment, as illustrated in Figure 3a. If the pathway was affected by the potential of nearby pathways, sometimes it revealed sharply folded segments (see Figure 3b). In this case, parametric fitting was easily applied to get a smoothly curved pathway.

### 2.2. Augmented Ground Truth and Input Image Generation

After constructing the pathway segments for the capillary network, the next step is to set the capillary diameter of the model according to the scale of real OCT image data. Then, both the augmented input images and their corresponding ground truth image data can be constructed.

For the augmented images, in order to mimic the lightened capillary segments seen in real OCT image frames, 2D Gaussian spots were pasted along the segments of pathways in the working domain (see Figure 2e). The 2D Gaussian spot function is expressed as

(5) $$f\left(x,y\right)=I_{c}\;e^{-\left[{\left(x-x_{0}\right)}^{2}+{\left(y-y_{0}\right)}^{2}\right]/\left(2\sigma_{c}^{2}\right)}\;$$

where $x_{0}$, $y_{0}$ are the discrete coordinates of points along the pathway segments,$\;\sigma_{c}$ is the spread width of the spot point, defining the diameter of the capillary lumen, and $I_{c}$ is the intensity of the simulated lightened capillary segments. Up to this point, en face simulated capillary segments seen in real OCT data were augmented. Binarizing the resulting images, we got the ground truth image data. To generate the corresponding input image data, Gaussian noise was applied to generate a background with the desired signal-to-noise ratio (SNR). Real FF-OCT images of living skin are rigorously influenced by granular speckle noise (also called multiplicative noise) due to the interference of backscattered signals from non-stationary human skin tissue, as well as multiple backscattering from other skin tissue structures with non-negligible reflectance. In order to simulate this kind of phenomenon, bright patches were randomly added to the monochromatic background. To do this, a Gaussian center point was randomly selected within the working domain, and new Gaussian spread width ($\sigma_{speckle}$) and intensity $\left(I_{speckle}\right)$ were utilized to model speckle noise, where $\sigma_{speckle}=k\sigma_{c},\;k>\;4$, and $I_{speckle}$ is around the magnitude of capillary intensity $I_{c}$.

In real FF-OCT images of skin in vivo, the visualization of capillaries is fragmented. In order to imitate this situation, the augmented images of capillary pathways were randomly fragmented, as illustrated in Figure 1b and Figure 2f.

### 2.3. Stacking to Form Image Volume of ACN Data

As described previously, the segmentation of the capillary network should come from a full volume of FF-OCT skin images. As a result, our augmented images were stacked together to form an image volume of ACN data, in which some pure background layers were interlaced to approach the realistic case. Then, in the next step, to assess the quality of the ACN data, we built a deep learning model based on the ACN data and tested the model performance by segmenting the capillaries from the real FF-OCT image volume.

Typically, FF-OCT scans provide a large image volume with several hundred en face frames, each with a resolution of 488 × 648 pixels. This size of the image volume is too large for training a deep learning model with high memory consumption and computing time. Thus, in this study, we aimed to generate a target volume with a size of 128 × 128 × 64 pixels to utilize memory efficiently and reduce the training time. To create 3D volumetric images, 2D images with dimensions of 128 × 128 pixels containing identical but randomly fragmented pathways were stacked together to form a sub-volume with about 20 layers, among which a few pure background layers were randomly inserted to simulate the case where there is no flow of red blood cells in some instances. This sub-volume represented a set of connected capillary network systems, as seen in real OCT data. Next, one or two sub-volumes of different capillary network systems were inserted into the target volume backgrounds, as shown in Figure 2g. This works for both ground truth and input images. To prevent ambiguity in the model training and capillary segmentation, the sub-volumes of capillary network systems were well separated and were not located at the top or bottom boundaries of the target volume.

All generated ACN data (consisting of both ground truth and corresponding augmented input images) were saved for reuse. The 8-bit unsigned integer data type was used for efficient storage space and to maintain good quality of the raw image data.

### 2.4. Training the Deep Learning Model

**Figure 4:**
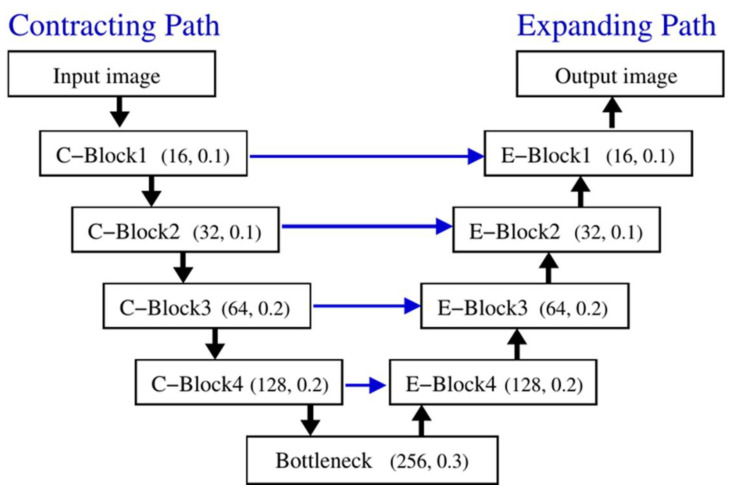
Structure of U-Net model. In each block, number of filters $N_{f}$ and dropout rate r used in convolution and up-sampling layers are labeled $\left(N_{f},\;r\right)$. Black arrows indicate direction of processing. Blue arrows indicate that extracted image feature maps of C-blocks are concatenated with corresponding E-blocks to enhance learning performance and maintain reconstructed image quality in expanding path. Refer to Table 1 for detailed structure of each block.

In order to validate our ACN data imitating FF-OCT images of skin capillaries, a deep learning model trained by the ACN data was tested on real FF-OCT data for capillary segmentation. In this work, the U-Net biomedical image segmentation model [40] was adopted and slightly modified for 3D image volume analysis. Briefly, the network consists of a contracting path for convolutional down-sampling (also known as the analyzer or encoder) to capture the context information, and an expanding path for deconvolutional up-sampling (also known as the synthesizer or decoder) to recover the spatial resolution with target labeling (see Figure 4) [41]. Each path consists of four major blocks. The detailed structure of these blocks is summarized in Table 1. In each block of the contracting path (C-block), the resolution of the image is halved with the extracted feature maps increased according to the number of the filters ($N_{f}$). In each block of the expanding path (E-block), the process is reversed by reconstructing the image with doubled resolution and labeled targets from the accumulated feature maps. In addition, to maintain the quality of the reconstructed image, the feature maps of the E-blocks are concatenated with those of the corresponding C-blocks to enhance the learning performance.

In order to process our volumetric image data for segmentation of capillary networks, the following modifications of the U-Net model were carried out. First, the third dimension was added to each filter of convolution, max-pooling, up-sampling, and stride to form a 3D cubic for each block of the contracting and expanding paths. This modification led to a volumetric shape of image data for both the input and output of the network, with the input dimensions of 512 × 512 pixels of the original U-Net model changed to 128 × 128 × 64 pixels. Second, padding was applied in each convolution and up-sampling layer in order to maintain the spatial size of the data. Finally, to tackle the problem of overfitting for the long training process, dropout and batch normalization techniques were utilized after each convolutional layer to stabilize the learning process.

The sample input and output image volumes for the U-Net model prediction are presented in Figure 10, images 1–16, and Figure 11, respectively. Figure 10 shows real FF-OCT data for model testing. In order to enhance the signal-to-noise ratio, the input images were averaged for every four frames to form a 128×128×16 image volume. Since our model performs predictions frame by frame, each frame does not interfere with the others, so the image volume fed into the model still has size 128×128×64, in which the first 16 frames contain real signals and the others are left blank. Figure 11 shows the prediction result of each input frame (except the last two, which obviously contain no data) of two models trained by different datasets. For more details, please refer to Section 3.4.

### 2.5. Evaluation Metrics

To evaluate the quality of our ACN images to represent in vivo FF-OCT data of capillary networks in human skin, we investigated two scenarios to assess the performance: (1) The trained model was first tested by the ACN volumetric testing datasets, which were not seen by the model during the model building stage. (2) After reaching the required accuracy level with the testing ACN datasets, the model was tested by real FF-OCT images of human skin in vivo with capillaries annotated by experts [14]. Both the ACN datasets and the real images were evaluated by the following reliable evaluation metrics. 

**Accuracy:** Accuracy is commonly used to assess the performance of pattern segmentation, which is defined as follows:

(6) $$A\;=\;\frac{TP+TN}{TP+FN+TN+FP}$$

where *TP* (true positive) and *TN* (true negative) are the number of voxels that are truly recognized as capillary (region of interest) and the background with respect to the ground truth, respectively, corresponding to the overlap of ground truth and segmented image. *FP* (false positive) and *FN* (false negative) represent the number of voxels of segmented images incorrectly recognized as capillary and background, respectively. Overall, the accuracy measures the positive (capillary) and negative (background) observations that are correctly classified over the total observations. 

**Precision, recall, and F1-score:** In addition to the above evaluation metrics, metrics of similarity measurement between ground truth and segmented images—precision (also known as reliability or positive predictive value), recall (also known as specificity or true positive value), and F1-score—were utilized. Here, precision (P) and recall (R) measure the ratio of a true capillary region with respect to the sum of true capillary and mis-identified regions, in which P counts the mis-identified voxels of capillaries and R counts the mis-identified voxels of the background. F1-score measures the harmonic mean of precision and recall. These metrics are mathematically computed as follows [14].

(7) $$P=\;\frac{TP}{TP+FP}$$

(8) $$R=\;\frac{TP}{TP+FN}$$

(9) $$F1=\;2*\frac{P\;*R}{P+R}\;$$

*P*, *R*, and F1-score are equal to 1 if ground truth and segmentation are exactly identical, and 0 indicates they are completely different. Finally, the overall performance of the model built from the ACN data was estimated from the average of these evaluation metrics.

## 3. Result and Discussion

This section presents the details of the automatic generation of ACN data, as well as the procedures of model building and real data testing to validate the quality of the ACN data. All codes were written in Python. The Keras 2.2.4 framework with TensorFlow version 1.14.0 backend on a Jupyter notebook version 4.4.0 was adopted as our developing environment for implementation of the deep learning model. Our computing platform was a desktop with Ubuntu 18.04 operating system, equipped with an Intel (R) Core™ i9-9900k 3.60 GHz CPU, two NVIDIA GeForce RTX 2080 GPU cards, and 64 GB memory.

### 3.1. Construction of Guiding Potential for Capillary Network

**Figure 5:**
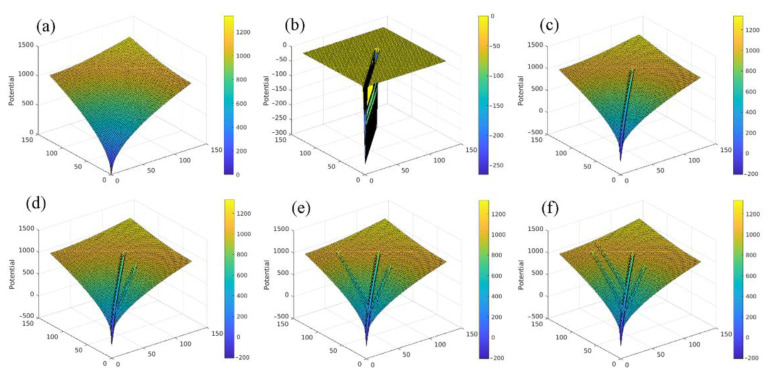
Illustration of development of potential field with respect to pathway network formation. (**a**) Root potential evaluated from Equation (1) based on root point located at (0,0). (**b**) Potential contributed by first pathway of Equation (2), forming a valley shape along the pathway. (**c**) Total potential, which is the sum of root potential (**a**) and first pathway potential (**b**). (**d**–**f**) Adding more pathways formed by other end points successively leads to the development of potentials contributed by these pathways one by one, which consecutively modify the total potential field.

The construction of the ACN data started by developing a guiding potential field on an en face two-dimensional working domain with a size of 128 × 128 voxels. First, the algorithm randomly selected a root point at the boundary and a few end points inside the working domain. As in the real FF-OCT skin image, the capillary network, seen in a specified visual region (around $200\times 200\;{\mu m}^{2}),$ revealed a limited number of branches; in our algorithm only three to six end points were generated. Then, starting from the root potential of Equation (1) formed by the root point, the pathways emerging from the end points and flowing along the direction of steepest decent of the total potential were formed one after the other. Whenever a new pathway was formed, it contributed the potential of Equation (2) and slightly modified the total potential. Thus, various forms of pathway branches and networks were constructed. Figure 5 presents an example of the development of the potential in the working domain for the root point located at the origin (0,0) with four randomly selected end points. As illustrated in the figure, the total potential field was slightly modified whenever a new pathway was formed, which then affected the shape of subsequent pathways. Some newly formed pathways can attach to previously formed ones and end up as branches or more complicated networks. 

**Figure 6:**
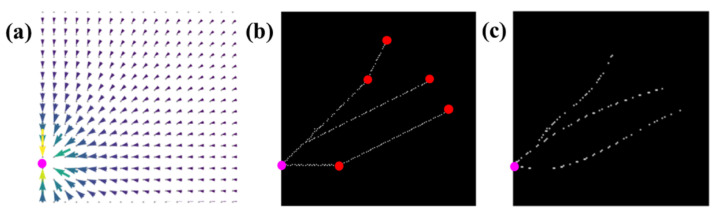
(**a**) Example of potential field of pathway network system in a quiver plot. (**b**) Constructed pathway network based on potential field (**a**). Pink dot represents location of root point; red dots indicate locations of end points. (**c**) Parametric fitting result to smooth straight segments of pathways.

Figure 6a presents another example of the potential field in a quiver plot, involving one root point and five end points. Figure 6b shows the pathway network constructed based on the potential field of Figure 6a. As seen in the plot, the pathway segments are straight and their attached points reveal sharp bending, which is far from natural to imitate real capillary pathways. Therefore, third-order polynomial parametric fitting was used to smooth the segments for more realistic capillary network shapes, as shown in Figure 6c. 

### 3.2. ACN Data Preparation and Model Building

**Figure 7:**
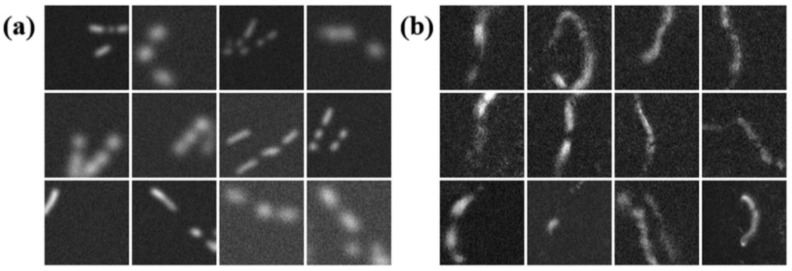
(**a**) Examples of augmented input images. (**b**) Examples of cropped capillary images from FF-OCT of human skin in vivo.

Figure 1b presents consecutive frames of ACN data images to simulate the in vivo FF-OCT human skin images with capillaries shown in Figure 1a. The parameters of the root and pathway potential used to generate Figure 1b are α=100, β=−20, and γ=−1 (see Equations (1) and (2)). To mimic the lightened capillary segments seen in the real OCT image, the parameters of Gaussian function Equation (5) for spreading the generated pathways are $I_{c}\in \left[0.05,\;0.08\right]$ and $\sigma_{c}\in \left[5,\;8\right],$ which define the lightened amplitude and diameter of the imitated capillaries comparable to those seen in the real OCT data, respectively. For the background noise, uniform Gaussian noise was first adopted, in which the signal-to-noise ratio was set to SNR∈[1,10] dB, and the variation of background noise was set to 0.125–0.5. Using the proposed algorithm together with the parameter settings given above, we can generate many kinds possible of augmented capillary network systems, as shown in Figure 7a. Compared to the real FF-OCT capillary images shown in Figure 7b, we tried to make the shape and contrast with the background of the augmented capillaries as similar to the real cases as possible. 

**Figure 8:**
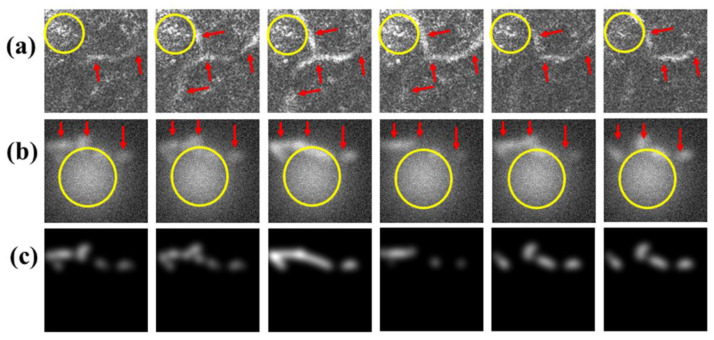
(**a**) Six successive en face FF-OCT images of human skin in vivo showing distribution of capillaries with speckle background and (**b**) six successive images of ACN data showing imitated capillaries with speckle noise in the background; speckle noise is indicated by yellow circle and capillary pathways by red arrows. (**c**) Corresponding ground truth images of (**b**).

Thus far, the background of the ACN data generated by Gaussian noise is smooth, which only mimics part of the phenomenon in real cases. However, in reality, skin tissue is rich in structures, which reflect more complicated patterns seen in FF-OCT images. For example, melanin granules are commonly found together with capillaries, which leads to significant speckles in FF-OCT images [14]. In addition, randomly backscattered signals by tissues also lead to random speckle noise. This speckle noise largely degrades the quality of image resolution and contrast [42]. This is a major obstacle when performing image processing, which makes capillary segmentation and detection harder [43]. Figure 8a presents an example of six successive en face FF-OCT images of human skin in vivo taken from a 25-year-old human subject, showing the presence of capillaries with speckled background. As demonstrated in Section 3.4, in the test of our first model trained by the ACN data without speckle noise, it did not perform well.

To address this problem, we tried to generate speckle noise on the ACN data, hoping that the performance of the model trained by these images could be improved. The generated speckle noise consisted of patches formed by the Gaussian function similar to Equation (5) and added to the smooth background (background with Gaussian noise), where the corresponding Gaussian width $\sigma_{speckle}$ was set to 8 times the spread width $\sigma_{c}$ of capillaries, and the amplitude $I_{speckle}$ was around the capillary amplitude $I_{c}$. Figure 8b presents six successive input images of the ACN data showing imitated capillaries with speckle noise in the background, with the corresponding ground truth images presented in Figure 8c. 

In order to assess the quality of our ACN data, a deep learning model based on U-Net was trained by the ACN data and tested on real FF-OCT data for capillary segmentation. In this study, 10,400 sets of volumetric images with smooth background and 7000 sets with speckle noise background were generated. Each volumetric image had dimensions of 128×128×64 voxels, and one or two sub-volumes (20 layers) of augmented capillary network systems were inserted, together with their corresponding ground truth datasets. Therefore, the deep learning model was trained by datasets with smoothed and speckled backgrounds, which were called model 1 and model 2, respectively. Then, the prediction power of both models was examined by testing the real FF-OCT dataset. To do this, the smoothed and speckled background datasets were divided into 7000 and 5200 sets for training, 3000 and 1400 sets for validation, and 400 and 400 sets for testing, respectively. 

In order to build a more stable and efficient model, we designed the following data sampling strategy for model training. In the beginning, all datasets for training, validation, and testing were generated and stored in a data pool. Then, during the training and validation steps of each epoch, a subset of data was randomly selected from the pool and fed into the model. There were 1000 and 300 randomly selected subsets in each epoch for training and validation for model 1, and 800 and 200 for model 2. Thus, in each epoch, the model only saw a portion of the total data pool, and each time the sampled datasets were different. This was to prevent overfitting, which is usually due to (not enough of) the same datasets repeatedly reviewed by the model so that the model can memorize the exact correspondence between the data and the ground truth. On the other hand, some datasets may still have a chance to be reviewed in different epochs, which can be helpful to enhance the training. Moreover, sampling a subset of data for training and validation in each epoch also prevents exhausting the limited amount of host and GPU memory while the total amount of datasets continues to grow. 

**Figure 9:**
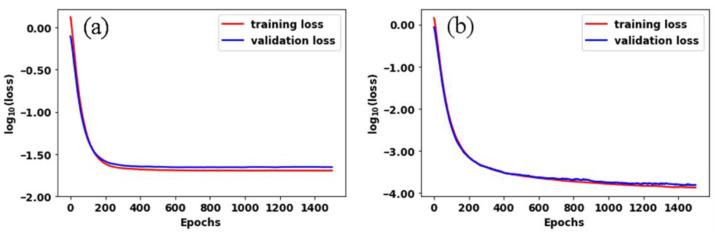
Learning curve of model training and validation using ACN data with (**a**) smoothed background (model 1) and (**b**) speckled background (model 2).

During the model training, a total of 1500 epochs with a batch size of 4, a learning rate of 0.0001, and adaptive moment estimation (Adam) were performed. Figure 9a,b shows the learning curves of training and validation losses over 1500 epochs for models 1 and 2, respectively. As vividly seen in the plots, the training and validation losses reasonably converge over epochs (decrease monotonically), and the validation is almost equivalent to that of the training. This indicates that our proposed models are free from overfitting.

### 3.3. Validation of ACN Data

To quantitatively analyze the quality of ACN data, four commonly used performance measures were implemented: accuracy, precision, recall, and F1-score. As mentioned previously, 800 sets of ACN data were generated independently for model testing, in which the 400 sets for model 1 were embedded with a smoothed background and the 400 sets for model 2 with a speckled background. These testing datasets were not investigated by the models until the models were well trained and ready for performance evaluation in the testing stage. With the assumption that our target, the capillaries, should occupy a small portion of the whole en face image, most of the areas should be the background and the noise should appear as variation around the background. Then, the prediction of these testing datasets by the models was binarized according to the following pixel value threshold in order to compare with the ground truth:

(10) $$p_{thresd}=p_{mode}+c\;p_{std}$$

where $p_{mode}$ is the pixel value of the largest portion of the whole en face image, which is regarded as the background, $p_{std}$ is the standard deviation of the all of the pixels, and c is a multiplier constant. In this work, we consistently set the multiplier constant as c = 2. As a result, pixel values larger than $p_{thresd}$ were identified as the foreground (i.e., capillaries), otherwise they were identified as the background.

Table 2 summarizes the results of capillary segmentation of models 1 and 2. These results clearly indicate that the proposed model achieves a good fit to the testing sets without overfitting for both smoothed and speckled backgrounds. Moreover, in all metrics, the scores of the model trained by the datasets with speckled background seem to be more uniform compared to those with a smoothed background, which indicates that model 2 is slightly better trained than model 1. 

### 3.4. Testing with Real In Vivo FF-OCT Human Skin Image

In order to assess the quality of our ACN data, the models well trained by the ACN data had to be tested on the real in vivo FF-OCT human skin image for capillary segmentation. Here, the real FF-OCT data were taken from [14], in which the in vivo FF-OCT images were acquired from the facial skin of a healthy 25-year-old male volunteer with informed consent, and the corresponding ground truth images were labeled by dermatologists. The size of the measured image volume was 291.60 μm×219.60 μm×109.24 μm, with pixel pitch of 0.45×0.45×0.193 μm/pixel. The detailed method of the FF-OCT experiment is explained in [13]. As an exploratory trial, a volumetric image with a size of 128×128×64 pixels was cropped from the raw image data for model testing to validate our proposed method. Since the ground truth annotated by the experts consisted of 2D images, the segmentation result from our 3D U-Net model had to be averaged over layers in order to obtain a 2D en face image and compared to the ground truth. 

**Figure 10:**
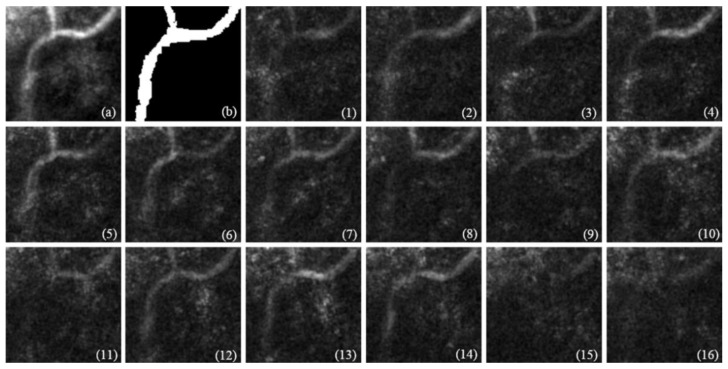
Real FF-OCT image used to test our models. (**a**) Total average of 64 frames of cropped volumetric image. (**b**) Expert-annotated ground truth. (1–16) are averages of every four frames of cropped volumetric image, forming 16 layers as input images for our models.

Since the contour of the capillaries of the real FF-OCT image was too fuzzy due to a small signal-to-noise ratio, before model testing, we averaged the images for every four frames in order to enhance our target versus the background, which resulted in an 128×128×16 image volume to input to our models. Figure 10 shows the real FF-OCT volumetric image used to test our models, together with the annotated ground truth image. Since it is obvious that the last two frames (Figure 10, images 15 and 16) have no signals, they were discarded from further analysis. Here, we see that even after averaging for every four frames, each layer still shows significant noise over the capillaries, and the pathways of the capillaries are variously fragmented, which leads to challenges in model prediction.

**Figure 11:**
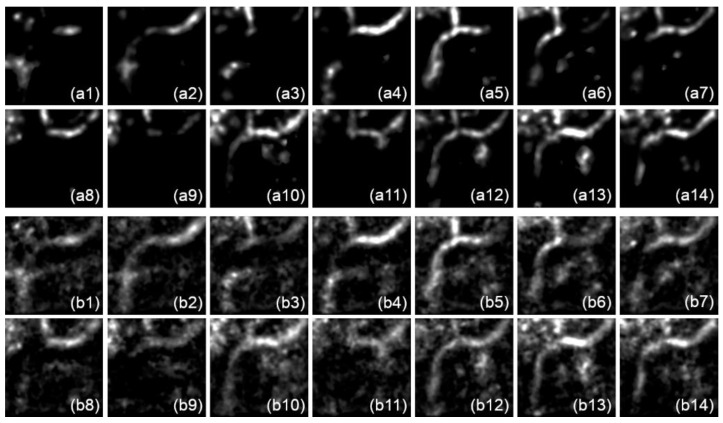
(**a1**–**a14**) Model 1 prediction and (**b1**–**b14**) model 2 prediction of Figure 10, images 1–14.

Figure 11 presents the prediction results of the input data in Figure 10 for models 1 and 2, in which each of the 14 frames of the input images (Figure 10, images 1–14) generated a corresponding predicted image, shown in Figure 11(a1–a14 and b1–b14), respectively. Comparing the results of the two models, we see that model 2 produced more signals and noise than model 1 in each predicted frame. This may due to the artificial patched speckle noise in the training data for model 2, which adjusted the model to try to extract as much information as possible from the noisy input data. Thus, the false positive rate of model 2 may increase significantly. On the other hand, the more foreground signal extracted by model 2 may also produce a higher true positive rate, as seen in the following analysis.

To assess the performance with the evaluation metrics, the prediction results of both models in Figure 11 had to be binarized to form 2D en face frames and compared with the ground truth pixel by pixel. To do this, the noise had to be suppressed as much as possible without affecting the real foreground too seriously. As described in the following section, we designed two binarization schemes for model prediction and compared their performance. 

#### 3.4.1. Simple Average with Threshold Binarization

In this scheme, the predicted frames in Figure 11 were averaged and a reasonable pixel threshold value was selected for binarization. Here, we set the threshold pixel value for binarization using Equation (10).

**Figure 12:**
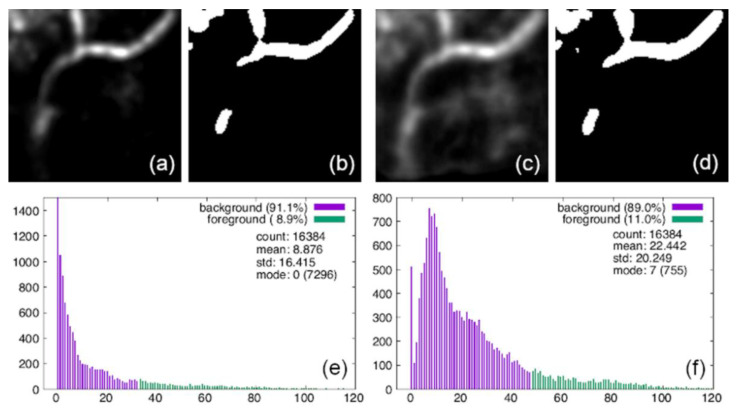
Binarization of simple average with threshold of both models. (**a**,**c**) Average of 14 frames predicted by models 1 and 2 (see Figure 11), with (**e**,**f**) their pixel histogram and statistics, respectively. (**b**,**d**) Binarization results of (**a**,**c**).

Figure 12 presents the binarization result of this scheme; Figure 12a,c shows the averaged en face image of the 14 frames predicted by models 1 and 2 (see Figure 11), respectively, together with their binarization results, shown in Figure 12b,d. The pixel statistics of Figure 12a,c used to set $p_{thresd}$ in Equation (10) are shown in Figure 12e,f, respectively, where their $p_{std},\;\;p_{mode}$ values together with the number of pixels at value $p_{mode}$ are listed. Comparing to the ground truth in Figure 10b, we see that in the binarized images most of the noise areas vanished, especially for model 2. However, the most apparent noise area shown in the upper left corner is still there in both cases, and the tailing capillary pathway extending to the lower left part of the image is suppressed in both predictions. In addition, the capillaries predicted by both models look almost the same, except that the one predicted by model 2 seems to be a little more coarse compared to model 1.

The elements of the confusion matrices and the performance metrics of the predictions compared with the ground truth for both models with binarization scheme 1 are summarized in Table 3 (rows labeled “bin-1”). The confusion matrices clearly manifest the portion mis-identified by the models. Both models give good true negative (TN) and false positive (FP) rates in the predictions, but not false negative (FN) and true positive (TP) rates. The high FN rate is due to the noise in the upper left corner, mis-identified as the capillary. The low TP rate is due to the missing tailing pathway of capillaries in the prediction. Furthermore, since model 2 has higher TP and lower FN rates than model 1, it has better performance metrics because of its coarser predicted pathway of capillaries.

#### 3.4.2. Counting Repeated Signals Binarization

Since the capillary patterns of the successive layers of an OCT image volume vary from frame to frame due to the discontinuous flow of red blood cells, the scheme of taking a simple average with a threshold may not faithfully represent all capillary pathways. Taking our testing data as an example (see Figure 10), the signal of the tailing pathway extending to the bottom left area of each en face frame is not apparent and clear enough. This leads to missed prediction in many of the frames by the models (see Figure 11). Thus, taking the simple average of predicted frames may suppress the intensity of this part too much to pass the threshold.

To resolve this issue, we propose another binarization scheme, which takes advantage of predicting each individual frame from the volumetric image. From the observation that each predicted frame contains different segments of capillaries, and many of them have an overlapping pattern among them, one can consider that the true signal (i.e., the capillary pathways) should be the patterns that appear several times in the predicted frames. We believe that this is a reasonable assumption, since the flow of red blood cells does not terminate during an OCT examination in a normal body in vivo. As a result, a merged en face image can be composed by investigating every pixel in all of the predicted frames to count how many times (N) it appears as the foreground. Then, this pixel is regarded as a true signal for N larger than a certain value, and its value is set to the maximum amid all predicted frames. The criterion for determining whether each pixel in each predicted frame is foreground or not is still Equation (10) with the multiplier constant c=2. Using this scheme, one can not only have a good chance to sample the true signal, but also to filter out randomly emerging noise.

**Figure 13:**
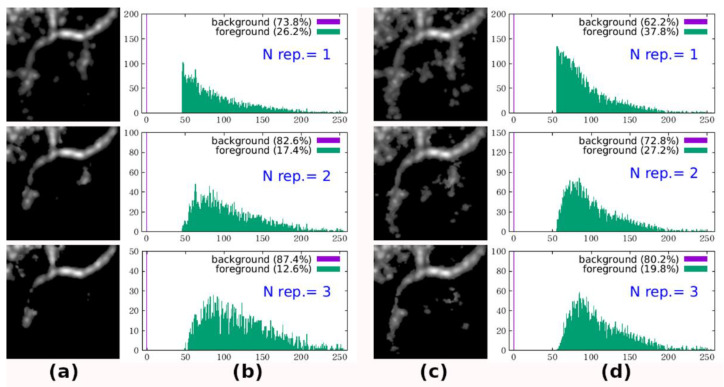
(**a**,**c**) Merged en face images of counting repeated signals for models 1 and 2, with (**b**,**d**) their pixel histograms, respectively; number of repeated signals labeled in blue text.

Figure 13a,c show the merged en face images of models 1 and 2, respectively, in which the results of the number of instances (N) of repeated signals of at least 1, 2, and 3 are presented. From the pixel histogram plots (Figure 13b,d), the background (pixel value 0) and foreground are clearly separated. Hence, we identify all pixels with a value larger than 0 as the foreground without additional criteria. Thus, the merged en face images can also be regarded as binarized images. As seen in the merged images, the larger number of repeated times N (which corresponds to the stricter criteria) suppresses more noise areas and parts of true signals, but the remaining true signal area is still larger than that using the simple average with threshold binarization; in particular, the missing tailing pathway is now apparent in this binarization scheme.

The elements of the confusion matrices and performance metrics of the predictions compared with the ground truth for both models are summarized in Table 3 (rows labeled “bin-2”). From the confusion matrices, the TP rate of model 2 with this scheme is significantly improved over that of model 1 and both models with the previous binarization scheme, but with some sacrifice in the TN rate. Furthermore, for model 1, this binarization scheme with N=3 almost recovers the result of the previous binarization scheme. The performance metrics also show outperforming results of model 2 with this binarization scheme for N≥2, excluding precision P due to the sacrificed FP rate. As a result, we conclude that model 2 using binarization by counting repeated signals with N≥2 is a viable method for performing capillary segmentation from FF-OCT data.

To this end, we demonstrated that our algorithm for generating ACN data to simulate real FF-OCT images with in vivo skin capillary networks can be effectively and efficiently used to train our model. It could significantly reduce the need for a large amount of effort to gather sufficient training data with experts’ annotations. Furthermore, the trained model could be used to perform capillary segmentation from the real FF-OCT data, with reasonable accuracy of ≥0.78, precision of ≥0.80, recall of ≥0.67, and F1-score of ≥0.75.

## 4. Conclusions

Regarding the importance of pathological diagonalization, clinical treatment, and histological study, angiography and capillary segmentation have become more crucial for investigation. FF-OCT provides a powerful tool for noninvasive in vivo 3D imaging. Nevertheless, conducting qualitative and quantitative analysis of capillaries from OCT data remains challenging and subjective. Moreover, building a good model to help with accurate capillary segmentation and analysis requires many well-annotated training data, which demand large-scale data collection and a great deal of effort by experts. In this study, we built a simulation algorithm to generate three-dimensional ACN data to mimic the capillaries seen in real FF-OCT skin images. A deep learning method was developed for automatic capillary segmentation to evaluate the segmentation performance of the generated images. To assess the quality of the ACN data, our model’s capillary segmentation ability was checked by an in vivo FF-OCT skin image for real testing. Our results suggest that ACN data could be used as an alternative to annotated real datasets. This significantly reduces the time and the amount of expertise needed for data collection and preprocessing, and is simple and powerful. Due to the difficulty in collecting more sets of real FF-OCT data with correct capillary annotation, only one set of real tests of our ACN data and models is presented. In the future, we will test our method with more widely annotated FF-OCT skin capillary images to confirm its robustness. In addition, as an exploratory study, the modified 3D U-Net model, together with the ACN data, provide a baseline tool for capillary segmentation in further applications, which are also worth studying and developing in the future.

This study adopted the conventional approach following the principles of pattern recognition and background subtraction, which is simple to implement. However, this method heavily depends on the quality of the input images. It is easily vulnerable to background corruption due to motion artifacts, variations in brightness, speckle noise, and the shadow effects of FF-OCT images. From the observation that the change in capillary patterns is much more significant than the change in background and noise over the layers of a volumetric FF-OCT image, more rigorous approaches, such as correlation mapping OCT (cmOCT) [28], robust principal component analysis (RPCA) [44], and short-time RPCA [14,15] could be used to separate the moving foreground (i.e., blood cells moving along the capillaries) from the nearly static background. These non-deep learning algorithms also show good accuracy for capillary segmentation, and in principle could give more promising results from input data with significant noise or a low SNR ratio. Thus, for further improvement of our proposed method, a deep learning model trained by our ACN data to segment out moving foregrounds will be highly beneficial. We note that, based on our proposed algorithm, our ACN data behaved similar to moving foregrounds over static backgrounds, which are ready for training this kind of model. Consequently, using deep learning models specially designed for fuzzy pattern recognition (suitable for biological structure segmentation) and incorporating temporal sequence tracing, such as recurrent neural networks (RNNs) [45] or the state-of-the-art mask RCNNs [46,47], would be interesting to investigate.

## Figures and Tables

**Table 1:** Detailed structure of each block of 3D U-Net model, where $N_{f}$ is the number of filters of corresponding blocks (see Figure 4).

Block Type	Layers	# of Filters	Filter Size	Stride Size	Padding	Activation
Contracting	Convolution	$N_{f}$	(3,3,3)	(1,1,1)	yes	ReLU
	Batch normalization	-	-	-	-	-
	Convolution	$N_{f}$	(3,3,3)	(1,1,1)	yes	ReLU
	Batch normalization	-	-	-	-	-
	Max pooling	-	(2,2,2)	-	-	-
Bottleneck	Convolution	$N_{f}$	(3,3,3)	(1,1,1)	yes	ReLU
	Batch normalization	-	-	-	-	-
	Convolution	$N_{f}$	(3,3,3)	(1,1,1)	yes	ReLU
	Batch normalization	-	-	-	-	-
Expanding	Up-sampling	$N_{f}$	(2,2,2)	(2,2,2)	yes	-
	Convolution	$N_{f}$	(3,3,3)	(1,1,1)	yes	ReLU
	Batch normalization	-	-	-	-	-
	Convolution	$N_{f}$	(3,3,3)	(1,1,1)	yes	ReLU
	Batch normalization	-	-	-	-	-
Outputs	Convolution	$N_{f}$	(1,1,1)	(1,1,1)	-	sigmoid

**Table 2:** Confusion matrices and performance metrics of averages over 400 testing datasets of models 1 and 2. TN, true negative; TP, true positive; FN, false negative; FP, false positive. A, accuracy; P, precision; R, recall; F1, F1-score. Mean and variation values of each quantity are average and standard deviation over 400 testing datasets.

Model	TN	FP	FN	TP	A	P	R	F1
Model 1 mean	91.38%	8.62%	4.39%	95.61%	0.997	0.915	0.955	0.929
Model 1 SD	5.49%	5.49%	5.04%	5.04%	0.002	0.100	0.063	0.049
Model 2 mean	99.98%	0.02%	5.80%	94.20%	0.998	0.994	0.935	0.964
Model 2 SD	0.03%	0.03%	2.54%	2.54%	0.001	0.007	0.028	0.014

**Table 3:** Summary of confusion matrix elements and performance metrics of both models tested by real OCT data (Figure 11) with two binarization schemes. bin-1 indicates simple average with threshold binarization; bin-2 represents counting repeated signal binarization, with results of 1, 2, and 3 repeated signals shown.

Model	TN	FP	FN	TP	A	P	R	F1
Model 1 bin-1	96.86%	3.14%	57.49%	42.51%	0.697	0.931	0.425	0.584
Model 2 bin-1	95.71%	4.29%	49.24%	50.76%	0.732	0.922	0.508	0.655
Model 1 bin-2, N rep. 1	81.85%	18.15%	26.01%	73.99%	0.779	0.803	0.740	0.770
Model 1 bin-2, N rep. 2	89.95%	10.05%	39.02%	60.98%	0.755	0.859	0.610	0.713
Model 1 bin-2, N rep. 3	94.30%	5.70%	46.68%	53.32%	0.738	0.903	0.533	0.671
Model 2 bin-2, N rep. 1	70.83%	29.17%	11.32%	88.68%	0.798	0.752	0.887	0.814
Model 2 bin-2, N rep. 2	81.16%	18.84%	23.44%	76.56%	0.789	0.804	0.766	0.784
Model 2 bin-2, N rep. 3	88.17%	11.83%	32.95%	67.05%	0.776	0.850	0.670	0.750

## Data Availability

The data presented in this study are available on request from the corresponding author.
